# A Review on Biofloc System Technology, History, Types, and Future Economical Perceptions in Aquaculture

**DOI:** 10.3390/ani14101489

**Published:** 2024-05-17

**Authors:** Bilal Raza, Zhongming Zheng, Wen Yang

**Affiliations:** School of Marine Sciences, Ningbo University, Ningbo 315832, China; balirana321@gmail.com (B.R.); zhengzhongming@nbu.edu.cn (Z.Z.)

**Keywords:** biofloc technology, aquaculture, sustainability, future economics

## Abstract

**Simple Summary:**

Biofloc technology reduces the environmental effects and reliance on fishmeal in the aquaculture business, which is experiencing growth. This technique removes organic nitrogen from aquaculture effluent, improves water quality, and generates microbial protein for use as a feed supplement for aquatic animals. Moreover, this method decreases the feed conversion ratio and manufacturing costs. Aquatic animals may obtain nutrients, fatty acids, and minerals from biofloc throughout the day. Biofloc, when combined with designed meals, offers a full food chain for aquatic animals, resulting in improved growth performance. In this review, we discussed the history and types of biofloc technology. We also reviewed microbial communities and factors related to BFT. Lastly, we described the advantages, applications, sustainability, and future aspects of biofloc technology.

**Abstract:**

Given the scarcity of water and land resources, coupled with the competitive nature of aquaculture, the long-term viability of this industry will depend on strategies for vertical development. This involves enhancing production environments, increasing productivity, and advancing aquaculture technologies. The use of biofloc technology offers a potential solution to mitigate the adverse environmental impacts and the heavy reliance on fishmeal in the aquaculture sector. This method is designed to effectively assimilate inorganic nitrogen found in aquaculture wastewater, thereby enhancing water quality. Additionally, this process produces microbial protein, which can serve as a viable supplemental feed for aquatic animals. Furthermore, this technique has the potential to reduce the feed conversion ratio, thereby lowering overall production costs. This article provides an overview of the evolving field of biofloc system technology within aquaculture. In this study, we will examine the historical development and various types of biofloc systems, as well as the factors that influence their effectiveness. Finally, we will explore the economic potential of implementing biofloc systems in aquaculture.

## 1. Introduction

Increasing awareness of environmental issues has led to a heightened need for ecologically sustainable management and cultural practices. Moreover, the extensive use of fish oil and fishmeal in aquaculture has placed significant pressure on the environment [1]. Water usage is another critical consideration [2]. During the 1980s, researchers recognized the importance of water exchange in mitigating numerous diseases, such as epizootics, in shrimp aquaculture farms [3]. Furthermore, the discharge of nutrient-rich effluent from intensive aquaculture systems into water bodies can contribute to eutrophication. This process has the potential to impact both the indigenous organisms in the ecosystem and the adjacent human activities [1,4]. As a result, several shrimp farmers have adopted measures to reduce water exchange, taking a more conservative approach to water usage [1].

Biofloc technology (BFT) represents an emerging alternative designed to promote environmentally sustainable methods in aquaculture production. The development of this technology aims to deliver both environmental and economic benefits by reducing water consumption and effluent discharges, decreasing dependence on synthetic feed, and enhancing biosecurity [3,5]. The utilization of BFT has been proposed as an environmentally responsible approach to aquaculture [5]. The concept of BFT has been recognized since the 1970s. However, significant research into the development and application of this technology has been conducted since the 1990s, yielding promising results [5,6]. According to the National Agricultural Library Glossary, published by the United States Department of Agriculture, biofloc technology involves the aggregation of algal, bacterial, or protozoan communities bound within a medium, along with particulate organic material [6]. This approach aims to enhance the quality of water, prevent diseases, and facilitate waste treatment within intensive aquaculture systems [7]. Biofloc represents a symbiotic phenomenon characterized by the coexistence of various aquatic organisms, heterotrophic bacteria, and numerous other species of microbes within the aquatic environment [5,7]. According to Browdy et al. [1], the elimination of ammonia from the culture system facilitates the recycling of waste materials into supplementary food sources for farmed aquatic animals. According to Crab et al. [8], it can be inferred that BFT has the potential to be a favorable option for promoting sustainable and ecologically conscious aquaculture practices. The term “biofloc” refers to a substance that is primarily composed of 60 to 70% organic matter, which includes a diverse mixture of microbes, including algae, fungi, rotifers, and protozoa. Additionally, it contains 30 to 40% inorganic materials, such as organic polymers, colloids, and dead cells [3,5]. According to Browdy et al. [1], these entities can reach dimensions of up to 1000 µm, exhibit irregular morphology, contain numerous pores, and facilitate the passage of fluids. The role of natural productivity in nutrient recycling and maintaining water quality is significant in BFT [9]. The consumption of biofloc by fish or shrimp has been demonstrated to offer numerous benefits, including an enhanced growth rate, reduced feed conversion ratio (FCR), and lower related expenses [10]. The promotion of growth has been attributed to the nutritional components of both bacteria and algae, potentially reducing the feed conversion ratio (FCR) by 30% through the utilization of biofloc [11]. It was noted that a substantial portion exceeding 28% of the daily food consumption for *L. vannamei* comprised biofloc [11,12]. Zaki et al. [13] reported that the utilization of feed in tilapia was more efficient with biofloc technology compared to fish raised in conventional water exchange systems. The nursery phase serves as an intermediate stage between the rearing of early post-larvae in a hatchery and the subsequent grow-out period [13,14]. Emerenciano et al. [15] found that incorporating bioflocs into the rearing environment resulted in a notable improvement in both the weight and final biomass of early larval shrimp. This enhancement amounted to approximately 50% and nearly 80%, respectively, compared to the standard pure water system [16]. The adoption of biofloc technology facilitates the advancement of intensive aquaculture practices while concurrently reducing the necessity for significant financial investments and ongoing maintenance costs [7]. Moreover, this technology provides the benefit of incorporating the capacity to recycle feed resources [4]. The approach is based on the principle of minimizing water exchange to bolster biosecurity measures and mitigate potential adverse effects on the surrounding environment. We will provide an overview of the expanding field of biofloc system technology in the context of aquaculture. In this study, we will also explore the historical background and various classifications of biofloc systems, along with the factors that influence their effectiveness. Lastly, we will investigate the economic prospects associated with implementing biofloc systems in aquaculture.

## 2. Biofloc Technology: A Brief History

### 2.1. A Brief History

The biofloc technology production system has emerged as a viable alternative to traditional aquaculture production methods, such as extensive and semi-extensive systems, which are typically used for cultivating commercially significant species like tilapia (*Oreochromis niloticus*) [7,17] and shrimp (*L. vannamei*) [7,18]. Additionally, it can serve as a valuable tool during the initial stages of cultivation, particularly in the early phases [8]. Biofloc technology originated in the early 1970s at the French Research Institute for Exploitation of the Sea (IFREMER) in Tahiti, French Polynesia [5]. Renowned researcher Gerard Cuzon played a pivotal role as one of the pioneers of this technology, collaborating with private corporations from the United States of America [5,7]. As a result, the application of shrimp farming expanded to include commercial operations, exemplified by those in Tahiti and the Sopomer farm [7].

Research on BFT for shrimp and fish farming commenced with the utilization of an active suspension system of microbes dubbed “microbial soup” [19,20]. Therefore, species of shrimp, like *P. sylirostris* and *L. vannamei*, were cultured using BFT in its early stages [5]. Initially, the concept of “heterotrophic” involved storing uneaten food, which contributed to the formation of a food web and the accumulation of feces in water bodies [9]. However, the recent iteration of BFT emphasizes the use of a carbon substrate and zero-water-exchange technology to minimize pollution [21]. Additionally, flour made from sorghum or wheat has been developed to enhance nitrogen removal efficiency [22]. Scientific research and pilot-scale trials were commenced at the Waddell Mari Culture Center in the United States of America in 1990, focusing on shrimp under the guidance of J. Stephen Hopkins. Similarly, finfish research was conducted by Yoram Avnimelech at the Technion-Israel Institute of Technology [5]. Two prominent research centers initiated a series of studies in 2000 that played a vital role in the progression of BFT technology in South America and North America. Under the leadership of Wilson Wasielesky, the research center at the Federal University of Rio Grande (FURG) in Brazil and the research center at Texas A and M University (Corpus Christi Campus) in the United States, led by Samocha and Tzachi, both concentrated their efforts on investigating shrimps [5,17].

In the late 2000s, the application of biofloc technology (BFT) expanded to include a wide variety of aquaculture species. These species include white leg shrimp (*L. vannamei*) [4,7], tilapia (*Oreochromis niloticus*) [23], channel catfish (*I. punctatus*) [24], giant freshwater shrimp (*M. rosenbergii*) [25], pink shrimp (*F. duoradum*) [26,27], red shrimp (*F. pauliensis*) [26], banana shrimp (*F. merguiensis*) [28], tiger shrimp (*P. monodon*) [29], Golden crucian carp (*Carassius auratus*) [30], Bocachico (*Prochilodus magdalenae*) [31], and African cichlid (*Pseudotropheus saulosi*) [32]. Among this group of animals, shrimp and tilapia stand out as particularly suitable for rearing using biofloc technology due to their efficient ingestion of biofloc as a protein source and their high adaptability to the biofloc system [26,30]. Until the 2000s, the acceptance of biofloc technology was limited due to the prevailing belief that clear water is more favorable for animal breeding compared to extremely turbid water containing biofloc [7]. A severe epidemic of viral shrimp disease prompted the widespread implementation of biofloc technology [33], including a variety of systems such as activated sludge or suspended bacteria-based systems, microbial floc systems, single-cell protein production systems, suspended growth systems, and zero-exchange autotrophic-heterotrophic systems [34]. 

The global dissemination of expertise in biofloc systems and the establishment of commercial farms have been facilitated by the human resources training provided by these organizations [6]. However, despite the advancements and advantages recognized by the academic and scientific communities, there is still room for the commercial expansion of BFT [7]. One contributing factor to this situation is the higher expenses associated with production and implementation, such as electricity costs, compared to conventional land-based culturing systems [19]. Furthermore, the supervision and execution of the technology are complex, requiring a higher level of technical expertise and continuous monitoring of water quality [8]. It is important to acknowledge that the implementation of BFT has primarily targeted aquatic organisms [8,12]. 

### 2.2. Fundamental Concept

Like recirculating aquaculture systems (RASs), cage farming, pens, and earth ponds, biofloc technology represents a type of aquaculture production system. The biofloc, comprising an assemblage of organic materials, serves as the fundamental functional unit of this system [5,27]. These clumps are composed of microorganisms such as heterotrophic and chemoautotrophic bacteria, cyanobacteria, archaea, viruses, microalgae, yeasts, and fungi [8]. Additionally, bioflocs can harbor or be inhabited by free-swimming invertebrates like rotifers, copepods, protozoa, cladocera, amoebas, ostracods, nematodes, and annelids [2].

Biofloc technology (BFT) is based on the principle of nutrient recycling through the enhancement of the carbon-to-nitrogen (C/N) ratio, which facilitates the growth of heterotrophic bacteria, algae, and other microorganisms within the system [6]. Biofloc serves three primary functions: regulating water quality, providing a nutritional supplement for cultured species, and engaging in microbial competition against pathogens [17]. The bioflocs consist of various constituents, including particles of organic matter such as unconsumed food, remnants of deceased organisms, excrement, suspended exoskeletons, colloids, and organic polymers [31]. These components, combined with microorganisms, merge to form conglomerates of varying dimensions, ranging from microns to millimeters [21,31]. Bioflocs are held together within a flexible matrix of polysaccharides, commonly referred to as mucus, which is produced by bacteria. Additionally, the occurrence of filamentous microorganisms or the electrostatic forces between the constituent particles contributes to the cohesion of the bioflocs [35]. According to Panigrahi et al. [36], bioflocs exhibit a higher density than water, leading to a relatively slow sinking rate of 1–3 m/h. 

BFT has been demonstrated to improve the performance of fish larvae and shrimp larvae [37] by improving hygienic conditions and reinforcing immune systems [37,38]. The implementation of limited or zero water exchange practices has been found to enhance biosecurity of farms and mitigate the transmission of diseases [39]. The microbial communities associated with BFT serve a dual purpose: facilitating the recycling of nitrogen compounds in water and offering protection against pathogens, such as AHPND in *L. vannamei* [40] and pathogens in tilapia [41]. Furthermore, these communities contribute to enhanced feed utilization and improved growth in cultivated organisms [31].

## 3. Types of Biofloc Systems

The term “biofloc” comprises many aquaculture production systems that rely on a combination of heterotrophic and autotrophic microbial activities to maintain the quality of water [7]. The essentiality of phytoplankton metabolism, specifically photosynthesis, and bacterial activities in this production system cannot be overstated [3]. A variety of biofloc systems have been developed, considering factors such as the farm’s location, the degree of agricultural intensity (super-intensive, semi-intensive, or intense), and the specific technical procedures implemented [5]. There are several distinct types of biofloc systems.

### 3.1. Without Media (SGS)

Without media SGS (suspended-growth systems) are alternatively referred to as the “algae, bacteria, zooplankton, and detritus (ALBAZOD)” systems [34]. The terms used by Hargreaves [34] include “photosynthetic suspended-growth aquatic system”, “organic detrital algae soup (ODAS)”, “zero exchange, aerobic and heterotrophic (ZEAH) culture system”, “aerated microbial reuse systems”, “activated sludge ponds”, “suspended, bacterial-based treatment process”, and “photosynthetic suspended-growth system (PSG)” [42]. The photosynthetic suspended-growth system is frequently utilized to produce significant amounts of microbial biomass. The system requires substrates, such as organic carbon sources, ammonia (NH_3_), and nitrite (NO_2_), along with vigorous aeration. This combination keeps the substrates and microbial communities suspended, thereby increasing the available surface area for bacterial activity [43]. According to Hargreaves [34], maintaining water quality is accomplished through the presence of a dynamic community comprising attached bacteria, phytoplankton, particulate organic matter, and other living organisms [34]. During this process, farmed aquatic animals consume phytoplankton, microbial flocs, and other related species, resulting in enhanced system efficiency and reduced production costs. The utilization of the green water culture system, commonly implemented in outdoor settings, represents an effective application of PSG technology [21,44]. Within this system, compounds such as NH_3_ and NO_2_ undergo oxidation processes facilitated by nitrifying bacteria that are cultivated on suspended organic waste. This oxidation ultimately results in the formation of nitrate [43]. Bacteria within the tanks consume the organic matter present as a source of nourishment [44]. Adequate aeration is crucial to sustain the microbial community, optimize the interaction between waste materials and bacteria, and enhance the output of phytoplankton output. Phytoplankton undergoes mortality and flocculation and, hence, needs the continuous removal of solid debris [44,45].

### 3.2. With Moving Media (AGB)

The technology known as attached-growth biofiltration (AGB) is alternatively recognized as an attached-growth membrane bioreactor (AGMBR) [17]. According to Qiao et al. [31], in this system, the transfer of substrates occurs from dedicated raising units to containers, facilitating the execution of a precise and predetermined operation. When comparing SGS and AGB, it can be observed that AGB is distinguished by its biofiltering media, which possesses a notably elevated specific surface area [31]. Hence, the efficiency of nitrification in aboveground biomass (AGB) is significantly greater than that in belowground biomass (SGS). Suspended solids can be reduced with this technique without sacrificing overall productivity [46].

### 3.3. Biofilm Reactor with Moving Bed

The Moving Bed Biofilm Reactor (MBBR) is a wastewater treatment method that operates under aerobic conditions. It relies on the utilization of plastic biocarriers as a substrate for the attachment and growth of microbial biomass [47]. The performance efficiency of the Moving Bed Biofilm Reactor (MBBR) is influenced by various factors, including the kind of media used in the reactor, the upper area of the biocarrier, the level of DO, and organic matter [47,48]. The MBBR is a sophisticated combination of the Submerged Growth Reactor (SGR) and the Attached Growth Biofilm (AGB) system [49,50]. By integrating the strengths of these two systems, the MBBR can attain a greater biomass concentration within the bioreactors. The Moving Bed Biofilm Reactor (MBBR) has demonstrated a high level of efficiency in the removal of biochemical oxygen demand (BOD) by up to 95% and chemical oxygen demand (COD) by up to 90% [47].

### 3.4. Periphyton Technology

The utilization of periphyton technology has been found to have multiple applications, including the removal of both inorganic and organic wastes, the natural augmentation of food production for cultivated species, and the improvement of water quality within culture [31]. In this technological context, stationary heterotrophic and autotrophic aquatic species, including fungus, protozoa, bacteria, phytoplankton, benthic organisms, zooplankton, and other invertebrates, are cultivated on submerged surfaces and utilized as naturally occurring nourishment for shrimp and fish communities [3,8]. The productivity of biomass of Periphyton is contingent upon the levels of light intensity and nutrient availability. In pond-based aquaculture, a healthy C/N ratio is essential for promoting and optimizing Periphyton production [40]. Periphyton is commonly employed in aquaculture practices to cultivate herbivorous, filter-feeding species of fish, omnivorous fish, crustaceans, and freshwater shrimp [3,25]. In aquaculture, the application of these specific biofloc technology system techniques have demonstrated various levels of effectiveness in the removal of ammonia and solids, creation of biofloc, and overall improvement of aquaculture outputs [51]. Membrane biological reactors (MBRs) and sequencing batch reactors (SBRs) have been identified as effective methods for the removal of sediments and nutrients from effluents of culturing farms [8]. In general, the inclusion of carbon sources is necessary for the operation of SBRs, but MBRs do not require carbon supplements. Furthermore, suspended-growth biological reactors (SGBRs) have been utilized to generate bioflocs derived from food processing waste [7,8]. Various types of reactors have been utilized for the purposes of treatment of wastewater as well as the elimination of ammonia from effluents of aquaculture. The technologies encompass fluidized bed reactors (FBRs), packed towers, and rotating biological contactors [48]. In the context of natural light exposure, aquaculture commonly employs two types of BFT systems: outdoor systems, which are exposed to natural light, and indoor systems, which lack natural light exposure [52]. 

The “green water” biofloc system, which is located outdoors, is widely utilized in industrial aquaculture [8,53]. This system comprises a combination of algal and bacterial communities that serve as a viable source of natural nutrition for aquaculture organisms [40,48,51,52,53,54]. Conversely, indoor BFT systems are typically operated within enclosed spaces, characterized by restricted illumination or even the absence of natural light exposure [55]. These systems primarily produce bacterial biomass, leading to the term “brown water” biofloc systems becoming popular [56]. Consequently, the extent to which farmed fish and shrimp assimilate bioflocs will mostly be contingent upon the specific biofloc system employed, the species and size of the cultured organisms, the size and density of the flocs, and the prevailing culture circumstances [27].

## 4. Composition of Bioflocs as Nutrient

Biofloc exhibits favorable nutritional characteristics. From a nutritional perspective, it has been observed that floc biomass has the potential to serve as a comprehensive nutritional resource, containing a wide range of essential nutrients and a diverse array of bioactive chemicals [10]. The nutritional composition of biofloc is influenced by various aspects, including the dietary preferences of the organism, its ability to consume and metabolize microbial protein, and the density of flocs present in the aquatic environment [35]. It has been found that shrimp, tilapia, and carp can effectively feed on single-cell protein, which is produced by heterotrophic bacterial populations through the assimilation of inorganic nitrogen [54]. According to Schveitzer et al. [57], on a dry matter basis, biofloc contains 39% protein, 4% fat, 7% fiber, 13% ash, and 18 kJ/g of energy. In their study, Serra et al. [58] conducted an observation on the composition of biofloc, which revealed the presence of 50% crude protein, 4% fiber, 2.6% crude lipid, 7% ash, and an energy content of 23 kJ/g. They also found that the feed quality employed in biofloc production had no effect on the biofloc quality, regardless of the crude protein content of the meal (35 or 22%) [58]. Ballester et al. [59] reported that bioflocs include 30.5% crude protein, 8.4% fiber, 4.8% crude fat, 39.3% ash, and 29.2% nitrogen-free extract.

There is variability in the protein content, ranging from 25% to 50%, and in the fat content, ranging from 0.5% to 15%, when expressed as a percentage of the dry weight [57,59]. According to Wasave et al. [14], biofloc can serve as a valuable reservoir of essential vitamins and minerals, with phosphorous being particularly noteworthy. Probiotics, which are bacteria intentionally introduced into the body to promote health benefits, such as *Lactobacillus* and *Lactococcus*, have comparable effects. The substitution of fishmeal or soybean with dried biofloc is observed in the diet [60]. According to the research directed by Schveitzer et al. [57], molasses was identified in biofloc, serving as a carbon source for the growth of *L. vannamei*, contributing to around 28.7–43.1% of protein content and 2.11–3.625% of lipid content. In contrast, cultivated tilapia fed with wheat flour exhibited 38% levels of protein and lipid levels ranging from 3.16% to 3.23% [23]. Organisms present in bioflocs contribute to the nutritional composition and ecological significance of the biofloc [50]. For instance, microalgae, which are a component of biofloc, can possess a protein content ranging from 30% to 65% of their dry weight [8]. Chlorophytes and diatoms exhibit a significant proportion of saturated fatty acids, ranging from 15% to 40% of the total fatty acid composition [44]. Conversely, green microalgae demonstrate a somewhat diminished presence of monounsaturated fatty acids, accompanied by elevated levels of polyunsaturated fatty acids [60]. The proximal composition of certain planktonic species seen in biofloc systems indicates that rotifers may possess crude protein levels ranging from 54% to 60%, whereas Cladocerans exhibit levels between 50% and 68%, and copepods possess levels of 70% to 71% [57].

In addition to these qualities, the carbon source type affects the digestibility and palatability of the grown species [59]. Overall, the best outcomes were obtained from bioflocs generated from glycerol [61]. Biofloc offers a comprehensive source of cellular nutrition and improves ingestion rate, nutritional absorption, and assimilation [10]. Brood stock diets enriched with biofloc improve reproductive success in *F. duorarum* and *L. vannamei* in terms of fecundity, spawning, and egg biochemical composition [61]. 

Research by Hamidoghli et al. [62] shows that bioflocs, which have a higher essential amino acid index, are abundant in amino acids like taurine and histidine. However, it was found that the limiting amino acids in bioflocs were arginine and lysine [63]. By recycling feed leftovers or recovering a portion of voided nutrients, the intake and renewal of bioflocs can enhance the effectiveness of the microbial population in utilizing feed [23]. The microbe enhances the growth rate, FCR, and weight gain of tilapia and shrimp while also eliminating excess nutrients [50,64]. Both the ability of bioflocs to maintain water quality in the BFT system and their nutritional value are influenced by the carbon source used in their production, even if they meet nutritional standards [57]. In addition to influencing the carbon–nitrogen ratio and promoting protozoa, bacteria, and algae, various carbon sources are also used to affect the composition of microbes and structure of biofloc community [59]. There was no need for water exchange when *L. vannamei* was cultured in a biofloc system supplemented with dextrose or molasses [65]. Emerenciano et al. [16] stated that because of high fat and protein content, bioflocs can be used as a biocontrol agent and as a source of natural, on-site nutrition for organisms in cultivation. They also treat feeding waste and lower ammonium concentrations in the system, which helps to sustain the water quality [66]. However, finding alternatives to fishmeal requires further research into the use of low-cost, fermented, non-traditional agro-industrial leftovers as carbon sources for the upgrading of waste into nutrient-rich feeds [14].

## 5. Species Cultured in BFT

Selecting the species to cultivate is the initial step in designing a biofloc system. Optimal results in biofloc systems are achieved when species capable of deriving nutritional benefits from directly consuming the floc are utilized [6]. The most suitable species for these systems are those that can tolerate high levels of sediment in the water and are generally resilient to low water quality [26]. Tilapia and shrimp, for example, exhibit physiological adaptations that enable them to efficiently assimilate biofloc and metabolize microbial protein, thus effectively using biofloc as a food source [17]. Carp, tilapia, and shrimp are commonly cultivated in nearly all biofloc systems [8,17]. Some species of shrimp are also cultivated in biofloc system (Table 1), such as *L. vannamei* [67,68,69], *L. stylirostris* [15,70], *Marsupenaeus japonicus* [38,71], *Litopenaeus setiferus* [72], *P. monodon* [29], *F. duoradum* [26,27], *F. pauliensis* [26], and *F. merguiensis* [28]. 

Researchers have undertaken numerous studies utilizing BFT (behavioral and functional traits) analysis on fish species belonging to the family *Cichlidae*, such as *O. mossambicus* [76], *Oreochromis aureus* [76], and *O. niloticus* [73,77]. Additionally, other commercially important fish groups extensively documented in the scientific literature include the *Cyprinidae* family, particularly *Cyprinus carpio* [78] and *Carassius auratus* [31]. Moreover, ongoing investigations are exploring indigenous fish species from South America, such as *Brycon orbignyanus* [18]. Recent research has focused on *Anguilla* spp. [79] and *Anguilla marmorata* [80]. The cultivated species in BFT exhibit common characteristics, including the ability to feed on suspended bioflocs and specialized morphological structures [6,17]. Tilapia, renowned for its effectiveness in aquaculture, especially in biofloc technology (BFT), possesses specialized structures known as *microbranchiospines* [81]. These structures facilitate water filtration and the trapping of bioflocs.

Commercial cultivation also encompasses the shrimp species *L. vannamei*. In a study by Kent et al. [82], electron microscopy was employed to analyze setae from third-degree maxillipeds. They suggested that juveniles possess the ability to select and ingest suspended food particles with diameters of approximately 10 µm, utilizing these net-like setae [82]. These shrimps demonstrate the capability to ensnare diatoms such as *Thalassiosira* and *Amphiprora*, both of which have diameters around 10 µm [73]. This capability helps to explain the remarkable adaptation of suspended biofloc systems. Additionally, other animals have shown efficient particle-capturing structures [82]. For instance, the freshwater shrimp *Macrobrachium rosenbergii* possesses anatomical features that allow it to effectively ensnare particles ranging from 250 to 1200 µm in size [25].

Because of its delicious flavor and strong market demand, shrimp has a high market value. Salinity, temperature, and dissolved oxygen levels are just a few of the physicochemical variables that this creature can tolerate [73,83]. It also has qualities that make it useful in aquaculture, including fast growth, high survival rates, and resistance to disease [39]. FAO [76] reported that Nile tilapia is one of the most frequently grown freshwater aquaculture fish species in the biofloc system. Barrıa et al. [81] reported that tilapia output reached 6.2 million tons in 2016. Consequently, it is one of the most common ways that people obtain their protein from animals [81]. In addition to its health benefits, tilapia is extensively consumed because of its low price and accessibility [84]. The product has the potential to be marketed in several formats, including gutted, live, or filleted, catering to the needs of wholesalers, intermediaries, hotels, restaurants, and other commercial enterprises [19]. Cultivation of tilapia has been encouraged because of the fish’s high market demand, rapid growth rate, robust physiological profile, and meat quality [85]. Despite their usual habitat being freshwater, tilapia has been shown to adapt to and even thrive in brackish and marine environments [81] due to its euryhaline status. For instance, across a wide variety of salinities, these fish show no fluctuation in the type of metabolic substrate that serves as their principal energy source [74].

Conversely, the reduction in water quality brought on by dense fish populations directly impacts the development and dissemination of diseases, causing stress for the fish and jeopardizing their physiological health [86,87,88,89]. The persistent issue of water pollution in tilapia production is caused by antibiotics, disinfectants, or any other chemical used to keep organisms alive [81]. Research has shown that overuse of antibiotics may result in drug-resistant tilapia-affecting bacteria as well as unintended consequences for the health of humans [13,14]. Additionally, higher stocking densities increase the likelihood of disease introduction into production systems and the importance of maintaining water quality in ponds and tanks through frequent water exchanges [13]. To sustain a natural ecosystem, where physical and chemical parameters such as salinity, pH, dissolved oxygen, temperature, and toxic metabolite concentration interact to maintain a stable and favorable environment for the development of fish and shrimp, the challenge of culture intensification is to maintain water quality characteristics that are comparable to those of a natural ecosystem [74,84]. The cultivation of tilapia has increased in recent years [85], and *L. vannamei* [72] in non-extensive systems based on biofloc technology. These systems are marked by concentrated aeration but minimal exchange of water, which lowers the use of antibiotics and increases animal density [20]. Nonetheless, understanding the nitrogen dynamics in such confined systems is crucial for avoiding disasters [90]. Species considered for biofloc technology must undergo evaluation based on their tolerance to suspended solids, nitrite, and low-to-moderate levels of ammonia nitrogen [42,43]. Additionally, the morphological structure of the candidate species should be conducive to effective biofloc grazing [7]. Therefore, specific fundamental criteria must be met by any candidate species to qualify for cultivation utilizing BFT [91].

## 6. BFT Microbial Composition

Over the years, various researchers have used different terms to describe BFT, including suspended-growth systems, microbial floc systems, active sludge, suspended bacteria-based systems, or zero exchange autotrophic-heterotrophic systems [17]. Ultimately, BFT operates by utilizing the microbiota within the culture system to maintain excellent water quality [3,8]. The fundamental idea behind this approach is to elevate the carbon/nitrogen ratio, primarily achieved by introducing an organic carbon source, thus stimulating bacterial growth to eliminate nitrogenous waste [6,17]. A biofloc is characterized as a particulate biomass-based medium abundant in organic matter and primarily inhabited by bacteria and other microorganisms [21]. The taxonomic composition can be maintained through the appropriate ratio of carbon to nitrogen [17]. The five phyla Bacteroidetes, Proteobacteria, Actinobacteria, Firmicutes, and Planctomycetes make up most of the taxonomic structure of bacteria, which are thought to be the most important components of the biofloc due to their abundance [75]. Other phyla have also been described, but to a lesser degree [55]. These include Tenericutes, Fusobacteria, Chlamydiae, Acidobacteria, Verrucomicrobia, Gemmatimonadetes, Armatimonadetes, and Nitrospira [75]. It seems that the initial colonizers of the biofloc could adhere to the substrate, assimilating its carbohydrates and thereby establishing the foundation for the potential introduction of other microbes that could fulfill various roles depending on the specific physicochemical conditions prevailing in the aquaculture system [89]. According to Panigrahi et al. [56], the biofloc fluctuating composition will eventually become stable upon reaching the mature phase.

Cardona et al. [70] used 16S rRNA amplicon sequencing to examine water samples from the biofloc system used to cultivate *Litopenaeus stylirostris*. The most abundant groups of bacteria are those belonging to the Bacteroidetes, Proteobacteria, and Cyanobacteria taxa [70]. Tepaamorndech et al. [92] used shotgun metagenomics analysis and 16S rRNA amplicon sequencing to define the complex of bacterial communities in the biofloc system growing *L. vannamei*. *Vibrio* sp. established 90% of the biofloc microbial population [92]. Other microorganisms included *Bacillus*, *Pseudoalteromonas*, *Lactobacillus*, *Acinetobacter*, *Photobacterium*, *Clostridium*, *Shewanella*, *Alteromonas*, *Pseudomonas*, and *Marinifilum* [93]. 

Using high-throughput sequencing, Wei et al. [35] examined the communities’ microbes within the ecosystem of biofloc and measured the 16S rRNA gene. The major biofloc bacterial taxa were likely members of the *Flavobacteriaceae* and *Rhodobacteraceae* families (*Marivita*, *Ruegeria*, and *Maribacter*) [35,94]. Ekasari et al. [25] recently analyzed the microbial community structure in *Macrobrachium rosenbergii* biofloc culture. It was shown that *Lactobacillus* species predominate in biofloc systems, with a 15:1 C:N ratio with *Enterococcus* species. De Sousa et al. [23] analyzed the microbial composition of grown-out biofloc used for the growth of genetically enhanced farmed tilapia. The composition of microbes of biofloc is comprised of 70% bacteria, 6% eukarya, 0.72% archaea, 0.17% viruses, and 23.45% uncategorized, according to the profile of metagenomic examined using the Illumina Nextseq500 platform shotgun sequencing [25]. According to additional classification, Caldilinea aerophila and Proteobacteria are two of the supreme prevalent genera in the biofloc microbiome [91]. Similarly, Yikai et al. [95] examined the bacterial composition within biofloc during the cultivation of *L. vannamei*. They found that Alphaproteobacteria predominated at 42%, followed by Gammaproteobacteria at 29%, and Bacteroidota at 27%. Collectively, these investigations demonstrate that the biofloc system dominant microbiota, such as *Bacillus*, *Lactobacillus*, and *Vibrio*, as well as other groups of bacteria, such as *Providencia*, *Halomonas*, *Pseudoalteromonas*, *Nitratireductor*, etc., may oversee producing advantageous effects on the environment, the host, and pathogenic microbes, in that order [60]. 

The functions of biofloc are intrinsically linked to the interactions of the microbial community in the spatial cohabitation of nutrient uptake and biochemical processes [96]. As natural bioremediation candidates, these communities play a vital role in converting nitrogenous waste products and maintaining water quality. Additionally, they are pivotal in forming nutrient-rich flocs that act as food sources, thereby enhancing population density nutritionally [97].

The many types of microorganisms in BFT exhibit intricate interactions that can be either complementary or competitive. Furthermore, a range of stimulatory and inhibitory interactions between algae and bacteria exists within the system [96]. The reason behind this phenomenon is that algae can release organic carbon compounds, which can vary from simple sugars to more intricate polysaccharides. These compounds are subsequently consumed by heterotrophic organisms. In addition, it should be noted that algae are organisms characterized by very brief life cycles [93]. Therefore, the decline in algae leads to an increased accumulation of organic carbon, consequently promoting the rapid growth of heterotrophic organisms [89]. However, research has demonstrated that bacteria can degrade organic substances, leading to the generation of crucial minerals, vitamins, and other bioactive compounds that have the potential to stimulate phytoplankton growth [3].

The presence of antagonistic growth chemicals, for example, antibiotics and allelopathic molecules, such as aponin, anatoxin, microcystin, and hemagglutinin, can lead to an inhibitory interaction between bacteria and microalgae [93]. According to Hargreaves [34], each group has the potential to alter the chemical environment of the other, with consequences for the other’s metabolic and nutritional processes. The lysis of microalgae cells can potentially be facilitated by bacteria through the synthesis of enzymes such as cellulases, glucosidases, and chitinases [97]. Substrate competition may also arise, depending upon temperature and ammonia levels, particularly in relation to ammonia or nitrate. According to Hargreaves [34], during the summer, phytoplankton tend to have a competitive advantage over nitrifying bacteria when it comes to low concentrations of ammonia-N. Conversely, in winter, nitrifying bacteria are more likely to employ a larger concentration of substrate to their advantage [46]. The bioflocs present in tanks that are predominantly inhabited by algae typically have a greenish hue and are primarily composed of filamentous microalgae, such as *Spirogyra*, *Anabaena*, and *Oscillatoria* [98]. These microalgae are typically loosely interconnected, resulting in a spatial arrangement within the tank [99]. According to Xu et al. [100], these organisms are subsequently distinguished by their low density/biomass and high settling volume output. Bioflocs mostly consist of nitrifying bacteria, which typically exhibit a greenish-brown hue. In comparison to bioflocs dominated by heterotrophs, these nitrifying bacteria-based bioflocs may exhibit a lower density [91]. Heterotrophic organisms typically dominate bioflocs where the largest densities are present. These bioflocs are characterized by a brownish color and a higher degree of aggregation [100].

According to Ebeling et al. [66], it was determined that the production of 15.80 g of algal biomass, 8.08 g of heterotrophic bacteria, and 0.25 g of nitrifying bacteria can be attributed to the presence of 1 g of ammonia. Furthermore, it has been observed that heterotrophic bacteria exhibit a higher growth rate compared to autotrophic bacteria [58]. Additionally, the amount of bacterial biomass produced per substrate by heterotrophic bacteria is reported to be 40 times higher than chemoautotrophic bacteria [93]. The microbial groups that are most prevalent have the potential to exert an impact on the process of nitrogen removal. The ability of phytoplankton and heterotrophic bacteria to remove nitrogen from the environment is the root cause of this phenomenon [91]. Conversely, nitrifying bacteria alone facilitates the conversion of noxious nitrogenous compounds into less harmful nitrate [89]. It can be suggested that heterotrophic-dominated biofloc technology (BFT) may exhibit enhanced nutrient conversion capabilities because of the fluctuating uptake of phytoplankton, which depends on exposure to daylight [6]. However, it is important to note that variations in nutrient removal by biofloc technology are typically observed mostly during the early stages of biofloc formation. Subsequently, nutrient removal tends to stabilize [3].

It can be posited that once a fully developed biofloc attains a state of balance within its diverse microbial population, these microorganisms collaborate synergistically to effectively eliminate nutrients from the culture media [7]. However, it is possible that systems mostly controlled by nitrifying bacteria or filamentous bacteria may contain a significant amount of less harmful nitrate [97]. Filamentous bacteria can accumulate nitrate within their cells, which can subsequently be released when DO levels are low [5]. In contrast, Zaki et al. [13] found that the crude protein (41.8%) and lipid (2.4%) of the biofloc-based system that was dominated by phytoplankton were both significantly higher than those of the bacterially dominated system (38.3% and 1.3%, respectively). According to Xu et al. [100], bioflocs consisting of a combination of bacteria and microalgae are more beneficial for shrimp cultivation compared to bioflocs dominated by heterotrophic bacteria. The enhanced development and feed consumption of shrimp performance cultivated in bioflocs containing a combination of bacteria and microalgae, as opposed to bioflocs dominated by heterotrophic bacteria, clearly indicates this phenomenon [93].

## 7. Factors Affecting Microbes during Floc Formation

The BFO community exhibits a rapid and efficient response to alterations in environmental conditions. While detecting these shifts may be challenging, they manifest through the activation or inhibition of pathways, as well as changes in the community’s composition, structure, and function [15]. Environmental alterations, including both biotic factors, such as predators, and abiotic factors, like salinity, can influence the generation, biodegradation activities, and abundance of BFOs [28]. Moreover, factors such as the farmed species, ambient conditions, food availability, and carbon supply can all impact the density of biofloc bacteria [25].

### 7.1. Salt Concentration

A change in salinity can influence the population density of heterotrophic bacteria and other BFOs. [26]. According to Maica et al. [101], chlorophytes exhibited dominance as the primary species of algae in environments characterized by low salinity. Conversely, diatoms were found to be the dominant species in environments with greater salinity levels [102]. Hosain et al. [103] observed comparable findings, indicating that chlorophytes exhibited higher levels of abundance at a salinity of 5 g/L, while diatoms were more abundant at a salinity of 32 g/L. Chlorophytes exhibit diminished nutritional value compared to diatoms because of their reduced quantities of crucial polyunsaturated fatty acids and suboptimal digestion by zooplankton and domesticated animals [16]. This phenomenon could potentially explain the observed decline in performance displayed by the species in the studied cultures due to decreasing salt levels and a consequent reduction in diatom abundance [103]. 

In their study, Hwihy et al. [73] identified salinity as the primary determinant of ciliate density. They observed that higher salt levels were associated with reduced ciliate abundance. Jiang et al. [21] also reported similar results. In their study, Maica et al. [101] observed a positive correlation between higher salinities and an increased abundance of diatoms and flagellates in biofloc shrimp culture. Conversely, they found that lower salinities were associated with higher numbers of chlorophytes and ciliates in the same system [104]. In their study, Khanjani et al. [72] examined the biofloc culture of pacific white shrimp and the effect of different salinity levels and food sources on the population density of heterotrophic bacteria. The findings revealed a positive correlation between salt levels within the range of 10–32 g/L and the density of bacterial populations [72].

### 7.2. DO Level

The diversity and composition of biofloc communities in culture systems are significantly affected by the levels of dissolved oxygen [105]. Adequate dissolved oxygen (DO) levels increase the metabolic activity of chemoautotrophic and heterotrophic bacteria, enabling them to decompose carbon sources including organic and inorganic compounds and facilitate nitrogen conversion [34]. According to Crab et al. [8], for both biofloc communities and the farmed species to thrive, dissolved oxygen (DO) levels must exceed 5 mg/L. Additionally, aeration serves to keep biofloc particles suspended, preventing them from settling at the bottom of the medium and thereby inhibiting the formation of anaerobic zones [17].

As described by Ebeling et al. [66], specific types of bacteria known as nitrite-oxidizing bacteria (NOB) and ammonia-oxidizing bacteria (AOB) convert ammonia into nitrite and subsequently into nitrate during the nitrification process. This process requires a consistent and ample supply of oxygen; otherwise, it would be hindered [8]. Therefore, it is essential to ensure adequate oxygenation and uniform mixing of the culture water to facilitate ammonia nitrification, promote biofloc accumulation, and enhance the performance of microbial communities [7,17]. However, excessive aeration can disrupt the aggregation of flocs and cause them to rupture [34]. Therefore, it is crucial to maintain aeration intensity at an optimal level to prevent the flocs from breaking apart while still promoting the nitrification process [8].

### 7.3. C/N Ratio and Organic Carbon Source

The impact of various carbon sources on bacterial populations in BFT systems has been extensively investigated by researchers [40]. Increased availability of carbohydrates leads to a predominance of heterotrophic bacteria within the biofloc communities [68]. Since aerobic microbes consume oxygen, introducing an organic carbon source into the water of biofloc ponds reduces the amount of oxygen available. This phenomenon can lead to severe harm or even death for sensitive species within the culture [100]. In their study, Deng et al. [2] investigated the effects of different carbon sources on the microbiota of biofloc technology systems by introducing cellulose, tapioca starch, and a mixture of both to the cultivation of herbivorous carp (Table 2). Mass sequencing revealed that Bacterophyta and Proteobacteria were the predominant phyla, regardless of the carbon source used [2]. It is important to note that these two phyla are significantly prevalent across various aquatic environments and aquaculture systems [2,8].

In a study conducted by Khanjani et al. [72], utilizing simple carbon sources such as starch and sugar resulted in an increase in heterotrophic bacteria populations compared to using complex carbon sources like barley, flour, and corn. However, it is crucial to consistently monitor the balance between the densities of BFOs and appropriate levels of dissolved oxygen (DO) [94]. According to Perez-Fuentes et al. [74], the levels of dissolved oxygen (DO) decreased significantly, dropping from 3.2 mg/L to 1–1.5 mg/L after introducing an additional 0.13 g/L of molasses into the system. While these changes might not be lethal, the potential adverse effects on the bacterial population, as well as the performance of shrimp and fish in biofloc technology (BFT), must be considered [28]. One potential approach for implementing BFT involves utilizing sources of organic carbon that are considered of low value by other processing units. For instance, glycerol, a by-product of biodiesel manufacturing, is being considered as a replacement [16].

### 7.4. Temperature and Light

The complex influence of temperature and light is evident. Numerous studies have been conducted on activated sludge samples to determine if there is a relationship between floc strength and morphology as a function of temperature [71]. According to the research conducted by de Souza et al. [69], flocs could be deflocculated at temperatures as low as 4 °C, compared to the higher temperatures of 18–20 °C typically required. This phenomenon is likely attributable to a decline in microbial activity within the flocs. In their study, Duan et al. [71] observed that elevated temperatures within the range of 25–35 °C led to the sludge bulking, characterized by a sludge volume index (SVI) of 500 mL g^−1^. This swelling was attributed to the overproduction of extracellular polysaccharides [104]. Based on the preceding information, it is reasonable to expect that the best outcomes can be achieved by maintaining the water temperature between 20 and 25 °C, where the floc volume index is roughly 200 mL g^−1^. A strong correlation exists between water temperature and the concentration of dissolved oxygen [21]. 

Light plays a significant role in aquaculture and directly impacts the performance and composition of the BFO community [5,43]. The abundance of phytoplankton is influenced by light conditions, which, in turn, cause fluctuations in the nutritional composition of biofloc [3]. The importance of these variables in aquaculture lies in the ability of cultured species to consume algae, hence promoting their development [21]. Research from various sources shows that cultivating *L. vannamei* and other photosynthetic organisms outdoors has a positive effect as compared to growing them indoors [55]. Additionally, the type of light source can also have an influence on the bacterial community within the culture of *L. vannamei* BFT. In their study, Fleckenstein et al. [106] found that the use of green lighting led to lower nitrite content when compared to the effects of red, blue, yellow, and white LED lighting. Jiang et al. [21] conducted a study wherein they identified beneficial bacteria, including the genus *Paracoccus*, in biofloc technology (BFT) systems exposed to light. Conversely, they observed the presence of harmful bacteria, including the *Leucothrix* genus, in the biofloc systems devoid of lighting [43]. Changes in temperature can affect the growth rates, food conversion efficiency, and mortality rates of the farmed species [21]. Additionally, the DO content and light conditions will also have an impact on the culture species. Hence, there exists a limited body of knowledge regarding the effects of varying light intensity, kinds, wavelengths, photoperiod regimes, and temperatures on BFT systems. Further research is necessary to deepen our understanding of these factors and their impacts on BFT [21].

### 7.5. Nutrition and Feeding

Feeds and nutrition are indispensable components in aquaculture endeavors [6]. The major percentage of biofloc operating expenses is accounted for by feed costs [8]. The efficacy of the feed is closely linked to the success of target crop production, encompassing both direct nutritional impacts and water quality implications. Current trends in the nutrition of shrimp and tilapia have been the subject of recent evaluations [5]. Considerable investigation effort has been devoted to the reduction of meal content in diets, given the limited availability and escalating volatility of meal prices [35]. Current aquaculture nutrition research is preoccupied with investigating the direct and indirect effects of unusual protein sources incorporated into diet alterations in dietary composition and availability, effects on gut microbiota and immunity, side effects from anti-nutritional agents, and so forth, are examples of such effects [6]. The natural productivity of organisms cultivated in biofloc systems can significantly contribute to the maintenance of nutritional balance. The growth-promoting potential of pond water as a whole and biofloc systems for cultivating fish and crustaceans has been demonstrated in numerous scholarly works [72]. Biofloc’s fatty acid composition has been examined at levels found in commercial feeds [16]. It has been demonstrated that shrimp can be cultivated in biofloc systems using diets devoid of supplemental vitamins with minimal growth inhibition [101]. Furthermore, it has been established that flocs comprise amino acids and essential minerals [20]. Therefore, biofloc has the potential to supply vital nutrients that either improve performance when combined with complete feeds or enable innovative approaches to formulations that are less expensive by capitalizing on the nutritional contributions of floc [20,28]. In shrimp biofloc-based systems, nitrogen conversion proficiencies from feed sources may be increased by 19 to 28% [43].

Ahmad et al. [4] found that the efficiency with which tilapia absorbed protein was greatly improved, with an increase from 30% to about 60%, while utilizing bioflocs. Incorporating waste nitrogen into the microbial biofloc and then reintroducing it to the target crop may considerably improve conversion efficiencies, leading to greater environmental sustainability and, maybe, increased profits [6]. Furthermore, it should be noted that alterations in the protein source or protein levels have a significant impact on the digestibility of the feed, thereby influencing the overall feed consumption [19]. In biofloc systems, the significance of these parameters becomes more pronounced when waste material undergoes either mineralization or assimilation inside the system [8]. It is evident that when stocking densities exceed the highest levels of extent, the primary factors influencing processes within the system are feed inputs [31]. As previously mentioned, ensuring an adequate supply of oxygen to meet the demands of both the microbial population and target crops in biofloc systems is a critical part of systems. The direct relationships between feed inputs and aeration needs in shrimp production systems have been modeled in several different ways [6]. The immediate impact of feed consumption efficiency can be observed in the density of biofloc, the need for microbial oxygen, and the formation of sludge. The literature presents two solutions for the biofloc system input feed management [5].

One approach involves the utilization of nutrient-rich, high-protein feeds containing easily digested components, combined with feeding techniques that prioritize the regulation of FCR [13]. The goal is to provide enough feed just below the maximum desired crop demand. Another approach proposes the incorporation of feeds with reduced protein content or combinations with grain-based supplements to endorse the development of heterotrophic bioflocs and facilitate the utilization of waste materials [27]. The choice of a feed preparation technique may be contingent upon the specific characteristics of the biofloc system [8]. In the context of shrimp cultivation in lower-density ponds and low-salinity systems, it is important to consider the potential risks associated with ammonia and nitrite toxicity [43]. If steps are taken to prevent the formation of anoxic organic material, these dangers may outweigh those due to high biofloc densities [6]. In this scenario, it is advisable to consider the utilization of meals with reduced protein content or the incorporation of increased carbon inputs [21]. 

Conversely, in higher salinity settings, the management of biofloc densities and waste accumulation becomes crucial as shrimp-raising densities are elevated [45]. In this scenario, the presence of elevated salinity levels contributes to enhanced system resiliency against ammonia and nitrite toxicity. Nitrification processes, which control ammonia and nitrite levels, have become increasingly important, lending credence to this theory [26]. Using diets with higher protein content and putting an emphasis on feed utilization efficiency, it is possible to achieve accelerated growth of the desired crop and minimize the generation of waste [72]. In a similar vein, the cultural practices and feeding methods employed in tilapia farming diverge significantly from those utilized in shrimp aquaculture [61]. This disparity arises from the fact that tilapia exhibit a greater propensity for consuming particulate matter derived from water columns containing biofloc, hence enhancing their feeding efficiency [84]. To design and manage biofloc system efficacies, especially at greater densities, feeding behavior and feed utilization efficiencies have complex interrelationships that must be considered, as well as the oxygen demand of the target crop and microbial community associated with the specific application of the technology [8].

### 7.6. Total Suspended Solids

Suspended solids play a crucial role in the breakdown of organic matter in biofloc technology systems and impact on water quality [107]. They also affect the abundance and diversity of biofloc organisms [108,109]. Numerous studies have revealed that removing suspended solids from water influences microbial communities, specifically decreasing the populations of bacteria, cyanobacteria, and rotifers. However, this process does not significantly affect chlorophytes, diatoms, and dinoflagellates [20,107]. These findings highlight the significance of management practices and their influence on microbial populations in biofloc technology (BFT) systems [109]. Increases in total suspended solids often lead to higher microbial biomass in biofloc technology systems, as microorganisms use the carbon and nitrogen from animal manure and fertilizers. For the cultivation of *L. vannamei*, the recommended levels of suspended solids are 10–15 mg/L [59]. Total suspended solid should be sustained between 240–350 mg/L, and turbidity should be kept within the range of 70–200 NTU [59]. Implementing integrated multi-trophic aquaculture (IMTA), which cultivates various species that utilize both suspended and dissolved residues in the system, can effectively manage TSS levels [110].

### 7.7. pH and Alkalinity

The BFT employs autotrophic, heterotrophic, and chemosynthetic bacteria that utilize alkalinity, leading to a decrease in both pH levels and alkalinity within the system [3]. Autotrophic bacteria consume a larger amount of alkalinity due to their absorption of a greater quantity of inorganic carbon [12]. Ebeling et al. [66] observed that heterotrophic bacteria require 4.8 g of dissolved oxygen, 15.2 g of carbohydrate, and 3.6 g of alkalinity to transform 1 g of ammonium into 8 g of microbial biomass, yielding 9.7 g of CO_2_ as a by-product. A decline in alkalinity will disrupt the balance between carbonate and bicarbonate ions in the culture system, leading to a reduced capacity to buffer pH changes. This may cause pH levels to drop or become unstable [73]. Any deviation from pH levels below 6.5 or above 9.5 will adversely impact not only the microbes within bioflocs but also the cultivated organisms [8]. 

The decline in pH and alkalinity levels might have damaging effects on the cultivated organisms [3]. For example, *L. vannamei* reared in BFT without any alkalinizing compound throughout the culture period exhibited the poorest survival rate, growth performance, immune responses, and feed utilization compared to systems where varying sodium bicarbonate levels were added to maintain pH levels of 7.6 and 8.1 [7,111]. Among these, the system maintaining a pH of 8.1 demonstrated the most favorable performance [111]. The selection of alkalinizing compounds can significantly affect the efficacy of BFT and the cultured organism’s performance. Martins et al. [112] discovered that sodium bicarbonate outperformed calcium carbonate in maintaining pH and alkalinity levels in BFT for *Oreochromis niloticus*. This superiority was further evidenced by the enhanced growth rate of the fish rearing in such systems. Additionally, both the carbon source and the C/N ratio can impact the rate at which pH decreases in BFT [111].

## 8. Advantages of a Biofloc System

The presence of diverse microbiota acts as a protective mechanism against the establishment and proliferation of any specific pathogenic species [6,99]. Certain microbiotas can induce non-specific immune responses in shrimp [113]. In their study, Abakari et al. [51] observed an increase in phenol oxidase activity, which serves as an immunological biomarker, following the introduction of carbon loading in a biofloc system. The biofloc system significantly reduces the rate of *Streptococcus* infection in tilapia compared to a clear water system [114]. Additionally, the decreased susceptibility of shrimp cultivated in a biofloc system to various lethal diseases, including infectious *myonecrosis* virus, white spot syndrome virus, and early mortality syndrome/acute hepatopancreatic disease, is widely recognized [33,40]. The research conducted by Wasielesky et al. [75] demonstrated the effectiveness of biofloc in reducing the incidence of white spot syndrome virus in Laguna, a region in southern Brazil. The use of biofloc is widely acknowledged as an effective strategy for mitigating the emergence of diseases in aquaculture [1]. The role of biofloc in alleviating the symptoms of early mortality syndrome (EMS) and acute hepatopancreatic necrosis disease (AHPND) was explored in a workshop held in Ho Chi Minh City, Vietnam, in December 2013 [40,115]. This disease led to significant financial losses, amounting to USD 1.26 billion for the shrimp industry in Vietnam in 2011, and continues to cause global annual losses of USD 5 billion [40,115]. The presence of intense aeration and agitation in a biofloc system results in the suspension of a significant number of solids originating from shrimp waste and unconsumed feed. Consequently, this process effectively mitigates the accumulation of sludge and sedimentation [116].

The microbiotas in a biofloc system actively scavenge both particulate and dissolved waste materials [20]. An example of this phenomenon is evident in the biofloc system, where nitrifying bacteria play a crucial role in eliminating ammonia, a harmful by-product often associated with intensive feeding practices [22,56]. This process is like a substantial in situ biological filtration system that continuously purifies the water. The implementation of biofloc technology in pond systems results in the efficient removal of organic wastes from the water, thereby minimizing or eliminating the need for water exchange [19,20]. This practice not only contributes to the conservation of water, a valuable and limited resource in various regions, but also enhances the biosecurity of the pond by significantly reducing the potential introduction of pathogens through water exchange [8,117].

Extensive documentation highlights the beneficial effects of bioflocs on the immune response and growth in aquaculture animals [7,8]. The utilization of bioflocs in aquaculture has been found to have a positive impact on the innate immune systems of cultivated species [118,119]. This is attributed to the diverse array of immunostimulatory effects provided by bioflocs, which effectively combat microbial infections. The cell walls of heterotrophic microbial organisms may consist of lipopolysaccharides, glucans, or peptidoglycans. The activation of non-specific immune systems by microbe-associated molecular patterns (MAMPs) has been observed to substantially enhance the immune response in cultivated organisms [6]. As demonstrated by Panigrahi et al. [56], the cultivation of *L. vannamei* in a heterotrophic biofloc environment resulted in notable improvements in immunological response. Specifically, total hemocyte count and prophenoloxidase activity were both higher in the experimental group than in the control group [120]. Bioflocs can accumulate a bacterial compound known as poly-β-hydroxybutyrate (PHB) [68]. Previous studies by Khanjani and Sharifinia [121] have reported that the presence of PHB in aquatic animals maintained in aquaculture systems can benefit from biofloc. This is because biofloc increases the efficiency of animal growth, improves the digestibility of their food, and enhances their resistance to bacterial diseases. Additionally, the microorganisms in biofloc have the capacity to positively influence the composition of the gut microbiota, leading to improved immunological responses and growth performance in the organisms [68,118].

Furthermore, the microbial organisms in biofloc include a variety of digestive enzymes and nutritional factors, such as proteases and amylases. These components can naturally facilitate the digestive process and enhance the absorption and digestion of food [44,122]. Consequently, this can lead to more efficient feed consumption and improved growth performance in the host organism [92]. For example, previous studies have indicated that *Bacillus* sp. plays a role in enhancing the nutritional status of hosts, particularly by providing vitamins and fatty acids [123]. Additionally, *Bacillus* sp. has been shown to improve the growth and survival rates of *P. monodon* post larvae and other aquaculture animals [29]. Zokaeifar et al. [123] presented evidence indicating that the presence of *Bacillus* sp. has a substantial impact on various aspects of shrimp physiology, specifically enhancements in digestive enzyme activity, immune response, growth performance, and resistance to infection of bacteria [123]. The findings of this study demonstrate that the presence of *Bacillus* sp., a beneficial microorganism in the system, has a significant impact on amylase and protease activity [123,124]. Consequently, this causes a significant increase in the final weight and weight gain of juvenile shrimps. Additionally, several studies have shown that administering *Bacillus* sp. can significantly enhance disease resistance and survival rates in *L. vannamei* juveniles after being challenged with *Vibrio harveyi* [125]. Subsequent studies conducted by Sadat et al. [113] have examined the benefits of *Bacillus* sp. on various aspects of *L. vannamei* aquaculture. These aspects include feed efficiency, growth performance, bacterial abundance, body composition, water quality, and immune response. The results indicate that when *Bacillus* sp. is administered to experimental units, *L. vannamei* shows significant increases in weight, specific growth rate, length, average daily gain, and feed conversion ratio compared to the control group [113]. The presence of advantageous bacteria has been found to enhance various aspects of *L. vannamei* post larvae, including water quality metrics, feed consumption, immunological response, and survival [126]. Panigrahi et al. [56] conducted a study wherein they observed that the addition of beneficial bacterial species, specifically *Bacillus* sp., to the culturing water of white leg shrimp led to an important enhancement of feed utilization. Furthermore, this supplementation led to improvements in various parameters, including weight gain, length, feed conversion ratio, and survival rates of the species of shrimps [37,68]. The study conducted by Chai et al. [125] involved the utilization of indigenous *Bacillus* sp. strains derived from the intestinal tracts of wild and healthy shrimp. The results show that *Bacillus* sp. has a big positive effect on the ability of *L. vannamei* to grow, its immune system, and its ability to fight off microbes that cause disease [125,126]. Moreover, the cultured animals exhibit an enhanced non-specific immunological response, as evidenced by significant increases in lysozyme, serum albumin, total immunoglobulin, serum protein, myeloperoxidase, and respiratory burst activity (Table 3) [118,125]. Collectively, these findings suggest that the presence of advantageous microorganisms within the biological system has the potential to enhance the development, immune reactivity, and disease resistance of cultivated organisms [113,124].

## 9. Disadvantages of a Biofloc System

One prominent limitation that is readily apparent is the elevated oxygenation levels and subsequent high energy expenditure required to maintain optimal conditions for both the shrimp and the microbiota [17]. The biofloc system cannot survive a power outage that lasts for more than a few minutes [18]. The establishment of the nitrifying bacterial community in a biofloc system may take up to four weeks. The anaerobic denitrifying process, which removes nitrate, also struggles in highly oxygenated conditions [8]. Intense bacterial nitrification typically results in decreased alkalinity and, consequently, lower pH levels. Therefore, effectively managing a biofloc system requires significantly more advanced skill sets and well-equipped research facilities [7,19]. Continuous monitoring of floc volumes measured by Imhoff cones, as well as oxygen, pH, alkalinity, ammonia, nitrite, and nitrate levels, is essential [72]. Consequently, it is imperative that future research endeavors prioritize the exploration of optimal strategies for managing BFT in culture ponds.

## 10. BFT Applications

The initial commercial uses of biofloc technology in shrimp farming were documented in Belize during the mid-1990s [8]. The cultivating ponds had an approximate area of 1.6 hectares and were observed to yield shrimp production ranging from 11 to 26 metric tons every cycle [5]. The proliferation of commercial shrimp farms utilizing both small-scale and large-scale biofloc technology approaches is currently observed in several countries like India, Indonesia, Thailand, Malaysia, South Korea, and China [17]. The recommended dimensions for a biofloc pond in commercial settings range from 0.1 to 2 hectares. To ensure adequate aeration and proper mixing of particles, it is advised to equip the pond with suitable aerators such as paddlewheels and aspirators [116]. Technological advancements frequently result in increased productivity while maintaining little impact on the environment [27]. The production intensity in BFT ponds surpasses that in non-BFT ponds, as evidenced by the enhanced growth and quality of tilapia cultured within the BFT system. BFT enhances output and productivity by facilitating the provision of superior fish juveniles, which is a crucial component in the production process. According to Ahmad et al. [6], the inclusion of biofloc technology in the culture system resulted in around a 45% increase in production and individual weight growth compared to systems without BFT. In Indonesia, around 20–30% of farmers use this technique, typically managing pond areas of 0.5–1 hectare. This adoption rate results in the production of over 30 metric tons per cycle. In addition, the utilization of biofloc systems can be employed in combination with other methods of production of food, leading to the establishment of integrated systems that aim to enhance productivity by generating a greater quantity of food and feed from a given land area while minimizing resource input [8]. BFT applications have the potential to enhance the propagative efficiency of aquacultural species and improve the resistance and resilience of larvae, contributing to the consistent provision of superior-quality seeds [5,8]. The sustainable methodology employed in biofloc technology relies on the cultivating of microorganisms within the culture medium, which is advantageous due to its minimal to nonexistent need for water exchange [31,72]. Reducing water usage in aquaculture operations can effectively mitigate the financial burden associated with water exchange, which has the potential to impose limitations on intensive aquaculture practices [5,6]. The biofloc, consisting of microorganisms, serves two primary functions [8]. First, it helps to maintain water quality by absorbing nitrogenous compounds and producing microbial proteins within the system. Second, it enhances the feasibility of aquaculture by improving protein utilization and reducing the reliance on commercial feed, thereby reducing overall feed costs [6,8]. The expenditure on feeds constitutes a minimum of 50% of the overall expenses in aquaculture production, mostly attributed to the elevated expenses associated with the protein constituent in commercially available feed [26,27]. According to Xu and Pan [68], the presence of the microorganisms linked with biofloc may have a beneficial impact on the digestive enzyme activity in shrimp. The incorporation of bioflocs into the diet at a BFT level of 75% has been shown to lead to enhanced growth performances and increased activity of digestive enzymes in the common carp [19]. Similarly, Anand et al. [29] described that the addition of microbes of biofloc as a dietary supplement at a concentration of 4% in the feed of shrimp can endorse the development and enhance the activities of digestive enzymes (Table 4). In recent times, bioflocs have emerged as a potential innovative technique for disease control due to their inherent probiotic properties, as opposed to conventional methods like the use of antibiotics, antifungals, and exogenous probiotics and prebiotics [7,8].

In intensive aquaculture practices, it is observed that natural food sources generally contribute to approximately 30–50% of tilapia growth when significant supplementary feed is provided [23]. Within a biofloc technology (BFT) treatment framework, tilapia exhibits a pronounced ability to efficiently utilize single-cell microbial proteins produced from total ammonia nitrogen (TAN) within the heterotrophic bacterial population. These characteristics render tilapia particularly suitable for cultivation using BFT methods [23,85].

## 11. Sustainability and Future Prospectus

Achieving sustainable development requires meticulous evaluation and integration of environmental resource management, social dimensions, and economic considerations. In the context of sustainable aquaculture’s future development, it is essential to consider the sector’s continuous growth and expansion. According to projections from the Food and Agriculture Organization (FAO), it is expected that aquaculture production will need to increase fivefold by the year 2050 [76]. This significant expansion must be carried out in a manner that is environmentally, socially, and economically sustainable [17]. Historically, aquaculture development planning from 1980 to 2000 did not place adequate emphasis on sustainability. During this period, shrimp farming was considered highly profitable, prompting the construction of new shrimp ponds. Unfortunately, these developments frequently occurred with little regard for their environmental impact [76]. Brazil leads in biofloc research in terms of publication volume, with China, Mexico, the United States, and India following [90]. However, Israel stands out for having the highest average citations per manuscript. The most cited publication in biofloc research, authored by Avnimelech from the Technion Israel Institute of Technology, has garnered 513 citations [130]. The species most frequently studied are prawns and tilapia, specifically *Penaeus vannamei* and *Oreochromis niloticus* [90,130].

Biofloc technology has proven to be an ecologically sustainable method for increasing the yield of cultivated species [6]. While significant advancements have been made in research over the past two decades, these improvements have not been equally reflected in commercial practices [17]. A comprehensive understanding of the biofloc ecosystem—including physiological and immune interactions, gut health, and microbial relationships—still requires further investigation [8].

Another area requiring further research is the determination of water quality tolerance levels for novel species cultured using biofloc technology [7,8]. Typically, tolerance levels derived from standard systems employing clear water or water exchange may not be applicable to organisms raised in biofloc systems, which feature minimal to no water exchange, high solids concentrations, and interacting microbiota [5]. Scientific investigations in this field are often conducted on a small scale under controlled laboratory conditions, which differ significantly from commercial environments [5,8]. Transferring and implementing technology on a larger scale is challenging due to the complexity of interacting components [8]. The fundamental differences between small-scale experiments and large-scale production could explain differences in results. Therefore, scaling up from experimental to commercial conditions is essential [17]. Conducting an economic analysis at the commercial scale is crucial to assess the feasibility and costs of implementing modules or farms [5,17]. Additionally, the high energy demands for adequate aeration, water circulation (to keep bioflocs suspended), pumping, and maintaining appropriate levels of solids significantly restrict the adoption of the biofloc technology system [8,9]. We should actively investigate alternative energy sources, such as wind turbines, solar panels, and biodigester gas, to enhance sustainability. Additionally, researching the genetic selection of species better suited to intense or super-intensive biofloc aquaculture is another promising area. Further research is also needed on disease resistance (e.g., against *Vibrio* species) and its impact on native species [27].

One potential area for further investigation is the optimization of carbon sources to enhance biofloc production [40]. Specific carbon sources can boost microbial populations, which, in turn, influence the nutritional characteristics of the biofloc and fish performance [43]. Consequently, it is essential to develop strategies for selecting and managing carbon sources to improve both the quality and quantity of biofloc. Managing microbial populations is another critical issue [18,19]. Disruptions in these populations can lead to the accumulation of harmful substances such as nitrate and ammonia, adversely affecting both fish well-being and water quality [43]. Therefore, it is crucial to regulate microbial communities and maintain a balanced ecosystem to effectively implement biofloc technology [17]. Additionally, effective monitoring tools are essential for ensuring stable and consistent production in biofloc technology systems [7,8]. Currently, the methods available for real-time monitoring of microbial communities and detecting changes in water quality are limited. Developing precise and reliable monitoring instruments is crucial for the successful implementation of BFT [7,17].

## 12. Conclusions

The growing adoption of biofloc technology principles worldwide has highlighted the inherent benefits of this system and encouraged further inquiries and research to address challenges and enhance effectiveness. A critical area of focus is the need for a deeper understanding of the complex microbial communities within biofloc systems, alongside the development of management strategies designed to guide and optimize their stability, formation, activity, and structural regulation. This issue is closely linked to concerns about water usage in the system and the potential for recycling water within and between production units. Further research is needed to better understand the factors influencing sludge production and management in biofloc systems, with the goal of optimizing the conversion of feed into shrimp or fish biomass while minimizing waste generation. Additionally, the study of pond and tank systems in terms of engineering and design is critically important, especially within the context of improving energy efficiency and reducing carbon footprints. The implementation of genetic selection programs has produced enhanced strains of shrimp and tilapia, demonstrating their potential for increased growth and the development of more resilient populations. The identification and incorporation of stocks with advantageous traits into biofloc-based systems could offer significant opportunities. As previously mentioned, prioritizing the development of specialty feeds, and improving feed management is critical, given their substantial economic implications and their impact on water quality and microbial community management. Further development and application of bio-economic models can help focus research efforts by establishing key production metrics for energy, water, and other resource usage. This approach will improve the effectiveness and sustainability of systems, considering both environmental and financial aspects. The future expansion of biofloc-based production methods is promising, as it offers significant opportunities for enhancing environmental sustainability and exploring new ways to reduce production costs, increase consistency, and boost profitability.

## Figures and Tables

**Table 1:** Main types of species cultured in BFT in aquaculture.

Cultivated Species	Country	Reference
*L. vannamei*	Brazil, China, Vietnam, India, Ecuador, Indonesia, Pacific coasts	[7,17]
*P. monodon*	China, India, Brazil, America	[29]
*L. stylirostris*	China, India	[15,70]
*Marsupenaeus japonicus*	China, Southeast Asia	[38,71]
*M. japonicas*	Southeast Asia	[17]
*D. labrax*	Turkey, Greece, Egypt, Spain	[11,17]
*S. maximus*	Turkey, France, Egypt, Spain	[7,17]
*T. rubripes*	Turkey, Greece, Spain	[5,17]
*Salmo salar*	Iran, Turkey, India	[11,17]
*E. awoara*	China	[35]
*O. niloticus*	China, Brazil	[73,74]
*C. gibelio*	China	[17,75]
*H. discushannai Ino*	China	[17,33]
*I. punctatus*	Vietnam, Thailand	[17]
*Macrobrachium rosenbergii*	China, India, Brazil	[17,25]
*F. duoradum*	India	[26,27]
*F. pauliensis*	Brazil, India	[26]
*F. merguiensis*	Southeast Asian countries	[28]
*Carassius auratus*	Vietnam, Thailand	[30,31]
*Prochilodus magdalenae*	America, Colombia	[17,31]
*Pseudotropheus saulosi*	India	[32]

**Table 2:** Use of different types of carbon sources for BFT in aquaculture.

Carbon Source	Species	C/N Ratio	References
Tapioca, molasses, tapioca rice bran, and by-product	*L* *. vannamei*	12 days	[2,10]
Molasses + dextrose + rice flour	-	First 5 days—15; 6–70 days—6	[10]
Dextrose	-	First 3 days—20; 4–30 days—6	[65]
Molasses	-	12, 15	[23]
50% molasses + 30% corn flour + 20% wheat bran	-	16	[28]
Wheat flour + molasses + starch	-	15	[63,66]
Molasses + palm sap	-	20	[11]
Corn flour, maida flour, gram flour, wheat flour, millet flour, rice flour, multigrain flour, and molasses	-	10–20	[21]
Glucose	-	15	[36]
Tapioca powder	*P. monodon*	12	[29]
Molasses	*L. vannamei* and *P. monodon*	-	[10,29]
Starch	*L. vannamei* and *M. rosenbergii*	10, 15, and 20	[21,25]
Molasses + wheat flour	*F. brasiliensis*, and *F. duorarum*	20	[2,27]
Wheat bran + molasses	*L. vannamei* and *F. paulensis*	20	[26,59]
Wheat flour and molasses	*O. niloticus*	8–11	[61]
Cellulose	*Tilapia*	11–16	[14]

**Table 3:** Effect of biofloc microbes on growth and immune response during aquaculture.

Microbial Species	Growth Performance	Immune Response	Disease Resistance	References
**Bacteria**				
*Lactobacillus delbrueckii* and *Bacillus pumilus*	Positive daily weight gain, specific growth rate	Positive myeloperoxidase, lysozyme, and respiratory burst activity	Positive survival against Aeromonas hydrophila challenge	[126]
*Aeromonas hydrophila Aeromonas salmonicida*, *Bacillus licheniformis*, and *Pseudomonas aeruginosa*	Weight gained and positive effect in growth	Positive effect of myeloperoxidase and lysozyme, respiratory burst activity	-	[92]
*Bacillus* sp. mixture	Weight gained and positive effect in growth	Positive expression of lipopolysaccharide prophenoloxidase, peroxinectin, and serine protein	Better survival (80%) than control (40%) against Vibrio harveyi infection	[123,125]
*Bacillus* sp.	Positive specific growth rate (SGR)	Positive respiratory burst, hemocyte count, and phenoloxidase activity	-	[91,124]
*Streptococcus*, *Staphylococcus*, *Bacillus, Neisseria* sp.	Develops bioflocs that assist in better growth performance	Positive immune response	-	[114]
**Microalgae +bacteria combination**				
*Schizochytrium* sp. and *Lactiplantibacillus plantarum*	Improved growth rate	Positive immune response	Resistance against pathogens	[4]

**Table 4:** Benefits of bioflocs in rearing the environment of aquaculture.

Benefits	References
Improved fish production	[13,24]
Better nutrition	[6,35]
Low FCR	[127,128,129]
Water quality within optimum levels	[3,14]
Fish health uncompromised	[14,73]
Alternative for intensification	[17,30]
Positive effect on digestive enzyme activity of shrimp	[37,92]
Improved growth performances	[124,125]
Natural probiotic effects	[64,91]

## Data Availability

Not applicable.
